# Derivation of consolidated normal reference values for right and left ventricular quantification by cardiac magnetic resonance using a novel meta-analytic approach

**DOI:** 10.1186/1532-429X-18-S1-O75

**Published:** 2016-01-27

**Authors:** Yang Zhan, Jaime L Shaw, Michael Chetrit, Michele Arcopinto, Russell Steele, Michael L Chuang, Warren J Manning, Jonathan Afilalo

**Affiliations:** 1 grid.14709.3b0000000419368649Jewish General Hospital, McGill University, Montreal, QC Canada; 2 grid.38142.3c000000041936754XBeth Israel Deaconess Medical Center, Harvard University, Boston, MA USA

## Background

Although cardiac magnetic resonance (CMR) has superior accuracy and reproducibility for quantification of ventricular size and function, its interpretation is limited by non-robust normative data derived mainly from small single-center studies.

## Methods

We performed a systematic review of the literature from 2003-2015 to identify all published studies that measured right and left ventricular parameters by CMR in healthy adult controls. The parameters of interest were: end-diastolic volume, end-systolic volume, stroke volume, ejection fraction, and mass (indexed to body surface area). Inclusion criteria were: SSFP-based acquisition and quantification on a short-axis stack using the method of discs. Exclusion criteria were: enrollment of non-healthy subjects, trained athletes, or overlapping data with other publications. Queries related to overlapping data were resolved by contacting the authors of the studies. We extracted age- and sex-specific data when available and stratified according to whether the papillary muscles were traced or not. We also accounted for the distribution of each parameter (rather than assuming a normal distribution), which was determined from a subset of individual patient-level data. Two observers reviewed the studies in duplicate and a third observer audited discordances. We performed a random-effects meta-analysis of the data (using the same approach developed for the American Society of Echocardiography chamber quantification guidelines) to generate pooled mean values for each parameter, upper reference values, lower reference values, and 95% confidence intervals surrounding each of these values.

## Results

Our PubMed search strategy identified 900 potentially relevant studies, of which 305 were eligible based on the pre-specified inclusion and exclusion criteria. The number of included studies ranged from 220 (N = 8,416 healthy subjects) for left ventricular ejection fraction to 28 (N = 993 healthy subjects) for right ventricular mass index. There were consistently fewer studies for right- as compared to left-sided parameters. There was a balanced proportion of males and females, and age ranged from 18 to 80 years. The normal reference values generated by our meta-analysis models are presented in Table 1 along with their 95% confidence intervals. When models were stratified by papillary muscle tracing, the only notable difference observed was for right and left ventricular mass index; as expected, mass was significantly greater when papillary muscles were included as part of the measured myocardial mass. Further analysis of age- and sex-specific differences in normative data was performed by meta-regression and will be presented.

**Table 1:** Normal Reference Values for Right and Left Ventricular Parameters by CMR

Parameter	Studies	Subjects	Lower Limit	Mean Value	Upper Limit
LVEDVI	123	5730	53 (51,56)	76 (75,78)	99 (96,101)
LVESVI	96	4831	15 (14,16)	28 (27,28)	40 (39,41)
LVSVI	70	4020	35 (33,37)	49 (48,50)	63 (61,65)
LVEF	220	8416	53 (53,54)	64 (63,64)	74 (73,75)
LVMI	127	6182	39 (36,41)	57 (55,59)	75 (72,77)
RVEDVI	65	2077	57 (53,61)	82 (80,85)	108 (104,111)
RVESVI	56	1688	20 (17,22)	35 (33,36)	49 (47,52)
RVSVI	39	1201	33 (30,36)	47 (46,49)	62 (59,64)
RVEF	83	2360	47 (45,48)	58 (57,59)	69 (67,70)
RVMI	28	993	13 (11,16)	20 (18,22)	27 (24,29)

* If papillary muscle were vs. were not traced, upper limit was 79 vs. 66 for LVMI and 35 vs. 19 for RVMI, respectively. Parentheses denote 95% confidence intervals surrounding point estimates. Abbreviations: LV, left ventricle; RV, right ventricle; EDVI, end diastolic volume index (mL/m2); ESVI, end systolic volume index (mL/m2); SVI, stroke volume index (mL/m2); EF, ejection fraction (%); MI, mass index (g/m2).

## Conclusions

The consolidated reference values generated by our meta-analytic approach have provided robust estimates of upper and lower limits of normal that reflect the accumulated body of published data for right and left ventricular quantification using SSFP-based CMR.

